# Predictors of the difficulty of transcervical subcarinal lymph node dissection for esophageal cancer

**DOI:** 10.1007/s10388-022-00983-w

**Published:** 2023-01-09

**Authors:** Hirotaka Furuke, Hirotaka Konishi, Hitoshi Fujiwara, Atsushi Shiozaki, Takuma Ohashi, Hiroki Shimizu, Tomohiro Arita, Yusuke Yamamoto, Ryo Morimura, Yoshiaki Kuriu, Hisashi Ikoma, Takeshi Kubota, Kazuma Okamoto, Eigo Otsuji

**Affiliations:** Division of Digestive Surgery, Department of Surgery, Kyoto Prefectural University of Medicine, 465 Kawaramachi Hirokoji, Kajii-Cho, Kamigyo-Ku, Kyoto, 602-8566 Japan

**Keywords:** Esophageal cancer, Transmediastinal esophagectomy, Subcarinal lymph node dissection

## Abstract

**Background:**

Transmediastinal radical esophagectomy (TME) is a new minimally invasive approach without thoracotomy. However, the transcervical dissection of subcarinal lymph nodes (SCLN) is challenging. The shape or narrowness of the mediastinal space, particularly around the aortic arch to the tracheal bifurcation, may increase the difficulty of this procedure. The present study aimed to clarify predictors of the difficulty of transcervical SCLN dissection.

**Methods:**

Patients who underwent TME between 2016 and 2019 were included (*n* = 126). Four indicators, the cervical angle, carina distance, aorta distance, and sternum distance, were defined as indicators of mediastinal narrowness by 3D-CT. The relationships between the difficulty of transcervical SCLN dissection and clinicopathological features, including the above indicators, were investigated.

**Results:**

In a univariate analysis, the cervical angle (*p* = 0.023), aorta distance (*p* = 0.002), and middle thoracic tumor (*p* = 0.040) correlated with difficulty. The median cervical angle and aorta distance were 15° and 33 mm in difficult cases and 19° and 43 mm in easy cases, respectively. In a multivariate analysis, the short aorta distance (odds ratio: 7.96, *p* = 0.002) and middle thoracic tumor (odds ratio: 3.35, *p* = 0.042) were independent predictive factors.

**Conclusions:**

The cervical angle, aorta distance, and middle thoracic tumor may predict the difficulty of transcervical SCLN dissection. In difficult cases, a transhiatal approach should be combined for complete SCLN dissection.

## Introduction

Esophageal cancer (EC) is the seventh most common cancer worldwide and the sixth leading cause of cancer-related death worldwide [1]. Esophageal squamous cell carcinoma (ESCC), the most common histology of EC in Japan and east Asia, shows extensive mediastinal spread from an early stage, and transthoracic esophagectomy (TTE) with extensive mediastinal lymphadenectomy has been the gold standard of radical surgery for ESCC [2]. Recent advances in endoscopic surgery have led to the development of transmediastinal esophagectomy (TME) [3]. TME achieves mediastinal lymphadenectomy similar to TTE, but with a lower frequency of postoperative pneumonia than open TTE [4, 5].

The transmediastinal approach provides an excellent view and handling along the esophagus for lymphadenectomy. In comparison with the transthoracic approach, the lymph nodes along the left recurrent laryngeal nerve or thoracic aorta, which are located behind the esophagus in the transthoracic approach, are dissected more easily with the transcervical approach using single-port mediastinoscopy [5, 6] or the laparoscopic transhiatal approach [7], respectively. In contrast, the lymph nodes located at the deep mediastinum, such as those along the aortic arch or tracheal bifurcation, require a high level of skill for sufficient dissection due to the complex anatomy and the farthest location from both the neck and abdomen. Subcarinal lymph nodes (SCLN) located along the tracheal bifurcation, including the bilateral main bronchi, are one of the regional lymph node stations to be dissected in radical surgery for ESCC [8, 9]. We initially used the transhiatal approach for SCLN dissection [7], but are now using transcervical approach according to the learning curve [10]. In comparison with the transhiatal approach, the transcervical approach reaches the subcarinal region with a markedly shorter distance and without cardiac compression for surgical field expansion. However, we sometimes encounter difficulties in transcervical SCLN dissection due to the shape or narrowness of the deep mediastinum.

The aim of this study was to identify predictive factors for the difficulty of transcervical SCLN dissection. Several angles and distances derived from the mediastinal shape were calculated by preoperative 3D computed tomography (3D-CT) and defined as indicators of mediastinal narrowness, and the relationships between the difficulty of transcervical SCLN dissection and clinicopathological features, including the above indicators, were investigated.

## Materials and methods

### Patients

A total of 126 patients who underwent TME and preoperative 3D-CT for EC at Kyoto Prefectural University of Medicine Hospital between 2016 and 2019 were retrospectively analyzed. TME was used as a primary option for curative resection. Patients on whom R2 resection was performed or SCLN dissection was skipped were excluded. 3D-CT was performed preoperatively for all patients undergoing TME to understand the transmediastinal surgical anatomy, particularly the mediastinal courses of the bronchial arteries [11]. Tumor stages and locations were classified according to the TMN classification of malignant tumors 8th edition [12], and the Japanese classification of esophageal cancer 11th edition [13], respectively. Treatment strategies were selected according to the Guidelines for the Diagnosis and Treatment of Carcinoma of the Esophagus [14]. Neoadjuvant chemotherapy (NAC) was performed for clinical stage II and III patients (5-FU + cisplatin or docetaxel + 5-FU + cisplatin). The present study was approved by the institutional review board (approval no. ERB-C-1414-1). Written informed consent on the treatments used and participation in the present study was obtained from all patients prior to treatment.

### Surgical procedure

TME was performed according to previously reported procedures [10]. In brief, the patient was placed in a supine position with both upper limbs fixed to the trunk and both lower limbs abducted. The left cervical procedure was initially performed using the single-port mediastinoscopy technique with a forced pneumomediastinum of 6–10 mmHg for upper mediastinal lymphadenectomy, including the lymph nodes along the left recurrent laryngeal nerve (RLN), which was followed by the right cervical procedure under direct vision for lymphadenectomy along the right RLN. Transhiatal esophagectomy with middle and lower mediastinal lymphadenectomy was then performed using a hand-assisted laparoscopy technique with a pneumomediastinum of 10 mmHg. Esophageal reconstruction was performed using a gastric conduit through the retrosternal route.

SCLN were basically dissected with the left cervical procedure. After esophageal dissection from the trachea, SCLN and the bilateral main bronchi were exposed on the dorsal plane or membranous portions, respectively. Then, SCLN were separated from the tracheal bifurcation. In case where it was difficult to separate SCLN from the tracheal bifurcation due to unsuitable device angle for dissection or peritumoral adhesion, SCLN were dissected safely and completely with the following transhiatal procedure as SCLN and bilateral main bronchi were already exposed transcervically.

All cases were operated by a dedicated team consisting of three expert surgeons, and the transcervical procedures were performed by the two surgeons who were certified as a specialist in esophageal surgery by the Japan esophageal society.

### Definition of the cervical angle, carina distance, aorta distance, and sternum distance

**Fig. 1:**
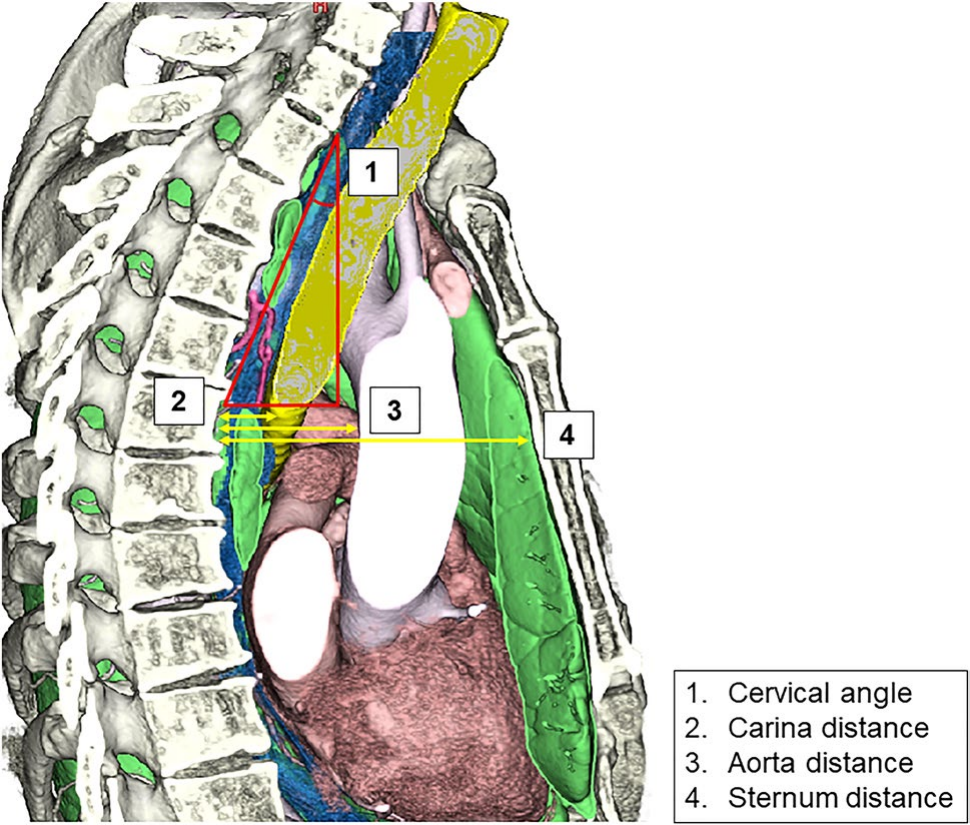
Indicators to evaluate the difficulty of SCLN dissection. The following 4 indicators were defined using preoperative 3D-CT images of sagittal sections through the carina: cervical angle (1), carina distance (2), aorta distance (3), and sternum distance (4). The details of these indicators were described in the “Materials and methods”

Four evaluation indicators were defined as follows using preoperative 3D-CT images of a sagittal section through the carina (Fig. 1). (1) Cervical angle: the angle calculated between the line of the body axis and the line connecting the lower edge of the first thoracic vertebra and the vertebra at the tracheal bifurcation level. (2) Carina distance: the length between the vertebra and the carina at the tracheal bifurcation level. (3) Aorta distance: the length between the vertebra and the ascending aorta or aortic arch at the tracheal bifurcation level. (4) Sternum distance: the length between the vertebra and the sternum at the tracheal bifurcation level. The cut-off values for these indicators in easy and difficult cases were calculated by the Youden index using ROC curve analyses.

### Evaluation of the difficulty of transcervical SCLN dissection

Patients in whom SCLN dissection was completed using the transcervical approach alone were selected as easy cases. Those in whom SCLN dissection was judged to be difficult due to unsuitable device angle for dissection or peritumoral adhesion, and completed with the following transhiatal approach were defined as difficult cases.

### Statistical analysis

Proportions or distributions of variables were compared using the chi-squared test. A multivariate analysis of predictive factors for difficulty was performed using a logistic regression analysis and odds ratio (OR), and the 95% confidence interval was subsequently calculated. Forward stepwise regression method was used for selecting independent and significant variables. All statistical tests were two sided, and a *p* value < 0.05 was considered to be significant. All statistical analyses were performed using JMP 10 software (SAS institute, Cary, NC, USA).

## Results

### Patient characteristics

The clinicopathological features of all patients are shown in Table 1. The median body mass index (BMI) was 18.9. Middle thoracic tumors were the most common (*n* = 53; 42%), followed by lower thoracic tumors (*n* = 33; 26%). All patients had squamous cell carcinoma. Sixty-four patients (51%) received neoadjuvant chemotherapy. The median cervical angle, carina distance, aorta distance, and sternum distance were 18°, 13 mm, 41.5 mm, and 96 mm, respectively. Fifteen cases (12%) were defined as difficult cases for transcervical SCLN dissection.

**Table 1:** Patient characteristics

Variables	Patients (*n* = 126)
Age, years	
Median	67 (41–81)
Sex	
Male	87 (69%)
Female	39 (31%)
Height, cm	
Median	164 (140–184)
Body weight, kg	
Median	56 (36–78)
BMI, kg/m^2^	
Median	18.9 (13.3–27.4)
Location^a^	
Ce/Ut	29 (23%)
Mt	53 (42%)
Lt/Ae	44 (35%)
NAC	
Presence	64 (51%)
Absence	62 (49%)
Blood loss, ml	
Median	153 (0–1500)
Total surgical time, min	
Median	340 (193–593)
Mediastinal surgical time^b^, min	
Median	122 (42–230)
Tumor size, mm	
Median	40 (3–190)
pT factor	
T0	5 (4%)
T1	54 (42%)
T2	16 (13%)
T3	44 (35%)
T4	7 (6%)
pN factor	
N0	67 (53%)
N1	41 (33%)
N2	15 (12%)
N3	3 (2%)
pStage	
I	48 (38%)
II	27 (22%)
III	44 (35%)
IV	7 (5%)
Cervical angle, degree	
Median	18 (6–32)
Carina distance, mm	
Median	13 (3–29)
Aorta distance, mm	
Median	41.5 (26–67)
Sternum distance, mm	
Median	96 (64–129)
Subcarinal lymph node dissection	
Easy case	111 (88%)
Difficult case	15 (12%)

^a^Ce, cervical esophagus; Ut, upper thoracic esophagus; Mt, middle thoracic esophagus; Lt, lower thoracic esophagus; Ae, abdominal esophagus

^b^Time used for the left cervical procedure with a single-port mediastinoscopy technique

### Representative 3D-CT images and surgical views

**Fig. 2:**
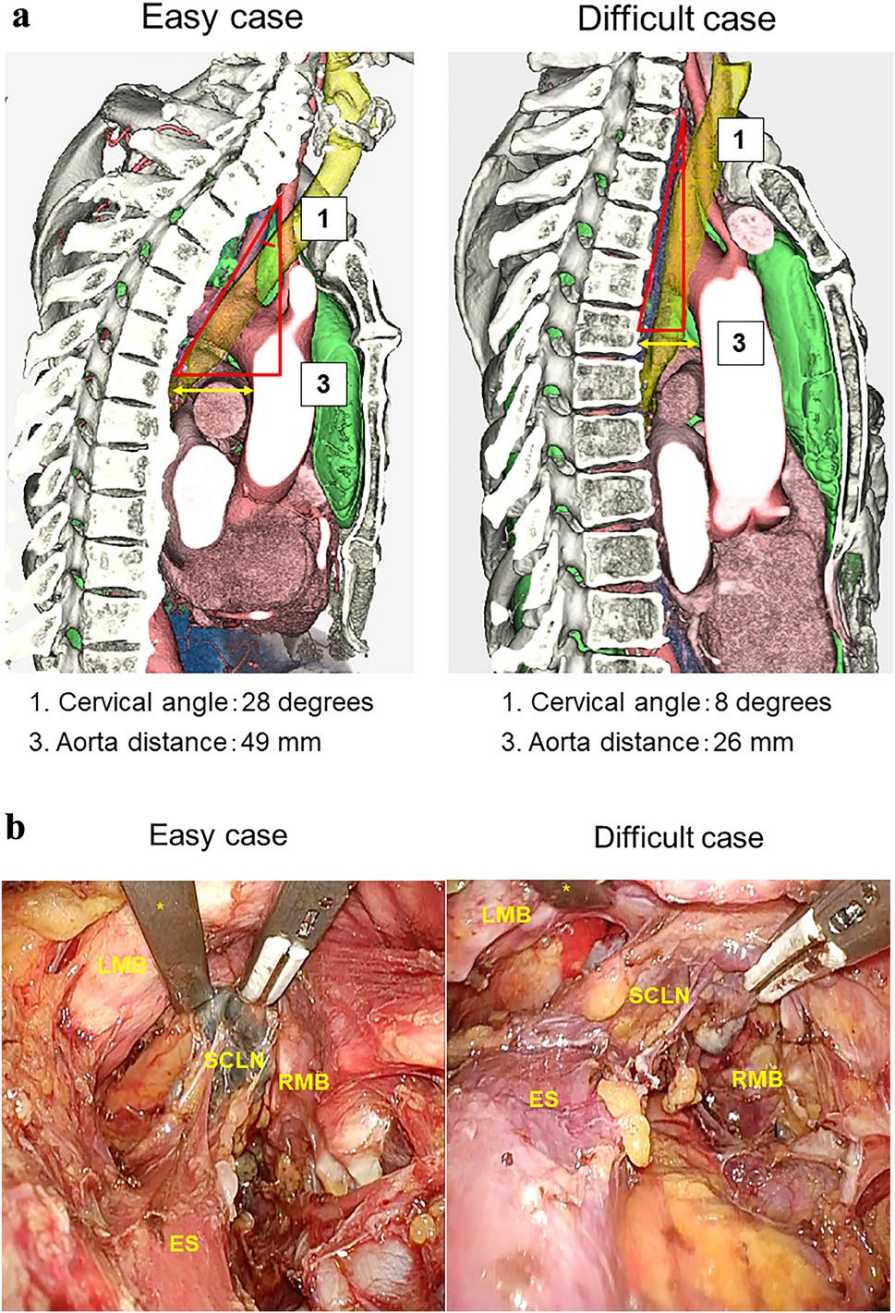
Representative images according to the difficulty of SCLN dissection. Preoperative 3D-CT image (**A**) and surgical view (**B**) in easy and difficult cases are shown. In the surgical view, SCLN (subcarinal and right main bronchial lymph nodes) stood up in a wide space between the tracheal carina and esophagus in an easy case, while in a difficult case, SCLN appeared to lie down in the narrow space. ES, esophagus; LMB, left main bronchus; RMB, right main bronchus; asterisk, left-hand retractor

Representative 3D-CT images and surgical views in easy and difficult cases of transcervical SCLN dissection are shown in Fig. 2A, B, respectively. The cervical angle was larger and the aorta distance was longer in easy cases than in difficult cases. In difficult cases, the median cervical angle was 15° and the median aorta distance was 33 mm. In easy cases, the median cervical angle was 19° and the median aorta distance was 43 mm (Table 2).

**Table 2:** Four indicators in difficult and easy cases

	Difficult cases (*n* = 15)	Easy cases (*n* = 111)
Cervical angle, °	15 (8–27)	19 (6–32)
Carina distance, mm	13 (3–23)	13 (5–29)
Aorta distance, mm	33 (26–55)	43 (26–67)
Sternum distance, mm	93 (78–129)	96 (64–127)

Median (range)

### Predictive factors for the difficulty of transcervical SCLN dissection

**Fig. 3:**
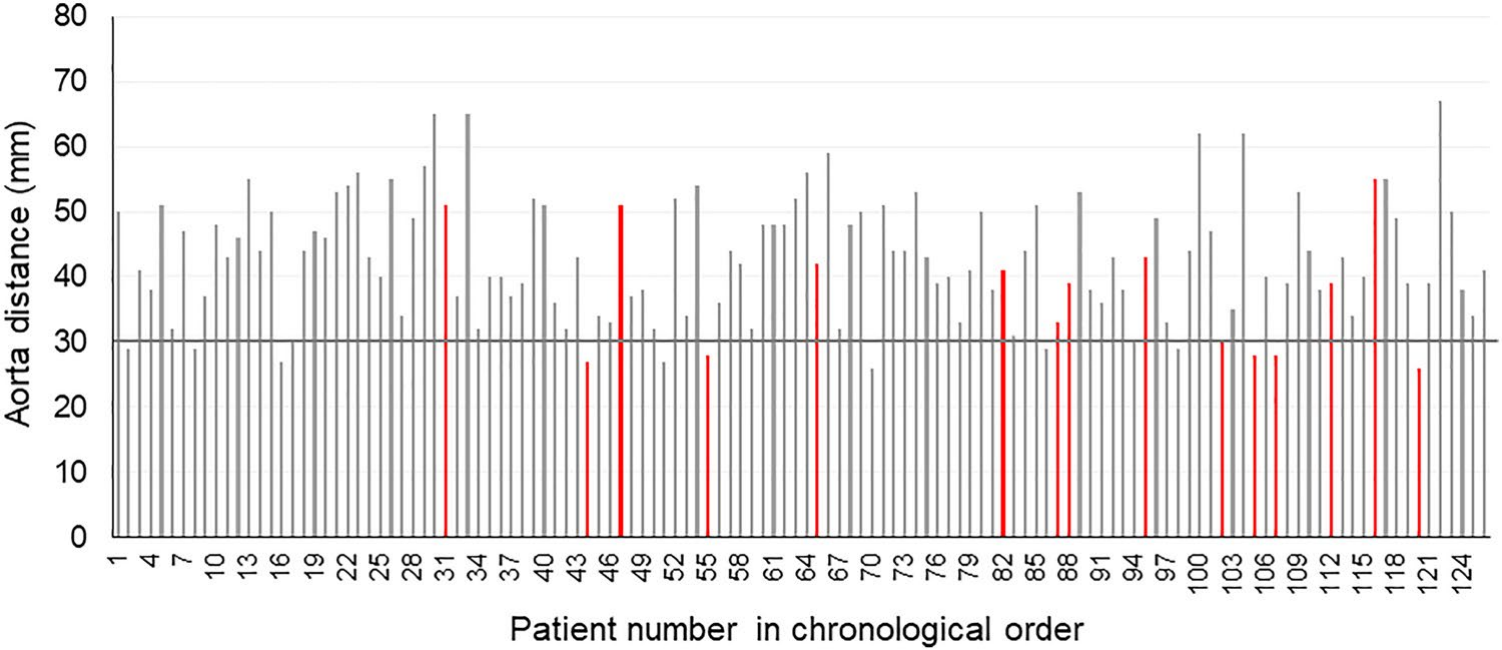
Chronological distribution of difficult cases in relation to the aorta distance. Difficult cases were highlighted in red, which were seen more in the latter half period (11/63, 17.5%) than in the former half period (4/63, 6.3%)

The results of univariate and multivariate analyses of predictive factors for the difficulty of transcervical SCLN dissection are shown in Table 3. In the univariate analysis, the tumor location (*p* = 0.040), cervical angle (*p* = 0.023), and aorta distance (*p* = 0.002) were identified as significant predictive factors, while BMI was also associated with the difficulty (*p* = 0.057). No significant differences in blood loss and mediastinal surgical time were observed between easy and difficult cases. In the multivariate analysis, short aorta distance ≤ 30 mm (OR 7.96, *p* = 0.002), and middle thoracic tumor (OR 3.35, *p* = 0.042) were independent predictive factors for the difficulty of transcervical SCLN dissection. In addition, chronological distribution of difficult cases was investigated in relation to the aorta distance (Fig. 3). Although the cases having the shorter aorta distance (≤ 30 mm) were seen almost equally between the former and latter half periods (7/63 and 8/63, respectively), difficult cases were more seen in the latter period (4/8, 50%) than in the former period (2/7, 28.6%).

**Table 3:** Predictive factors for the difficulty of subcarinal lymph node dissection

Variables (*n* = 126)		Univariate					Multivariate		
		Difficult cases (*n* = 15)		Easy cases (*n* = 111)		*p* value	OR^a^	95% CI^b^	*p* value
Age, years									
**> **70	48	3 (20%)		45 (41%)		0.109			
**≤ **70	78	12 (80%)		66 (59%)					
Sex									
Male	97	11 (73%)		86 (77%)		0.724			
Female	29	4 (29%)		25 (23%)					
Height, cm									
**> **160	80	9 (60%)		71 (64%)		0.765			
**≤ **160	46	6 (40%)		40 (36%)					
Body weight, kg									
**> **56	59	6 (40%)		53 (48%)		0.571			
**≤ **56	67	9 (60%)		58 (52%)					
BMI, kg/m^2^									
**> **20	79	6 (40%)		73 (66%)		**0.057**			
**≤ **20	47	9 (60%)		38 (34%)					
NAC									
Presence	65	9 (60%)		56 (51%)		0.485			
Absence	61	6 (40%)		55 (49%)					
Location									
Mt	53	10 (67%)		43 (39%)		**0.040**	3.35	1.04 – 12.2	**0.042**
Others	73	5 (33%)		68 (61%)			1		
Blood loss, ml									
**> **150	63	5 (34%)		58 (52%)		0.169			
**≤ **150	63	10 (66%)		53 (48%)					
Mediastinal surgical time, min									
**> **120	63	8 (53%)		55 (49%)		0.783			
**≤ **120	63	7 (47%)		56 (51%)					
Tumor size, mm									
**> **40	61	8 (53%)		53 (47%)		0.684			
**≤ **40	65	7 (47%)		58 (53%)					
pT factor									
T3–4	51	8 (53%)		43 (38%)		0.284			
T0–2	75	7 (47%)		68 (62%)					
pN factor									
N1–3	59	7 (47%)		52 (47%)		0.989			
N0	67	8 (53%)		59 (53%)					
pStage									
II–IV	78	11 (73%)		67 (60%)		0.491			
I	48	4 (27%)		44 (40%)					
Cervical angle, degree									
**> **15	84	6 (40%)		78	(70%)	**0.023**			
**≤ **15	42	9 (60%)		33 (30%)					
Carina distance, mm									
**> **10	81	11 (73%)		70 (63%)		0.426			
**≤ **10	45	4 (27%)		41 (37%)					
Aorta distance, mm									
**> **30	111	9 (60%)		102 (92%)		**0.002**	1	2.15–29.8	**0.002**
**≤ **30	15	6 (40%)		9 (8%)			7.96		
Sternum distance, mm									
**> **90	78	11 (73%)		67 (60%)		0.320			
**≤ **90	48	4 (27%)		44 (40%)					

Values in bold font indicate statistically significant differences (*p* < 0.05)

^a^OR, Odds ratio

^b^95% CI, 95% confidence interval

## Discussion

We recently reported that SCLN were dissected equally between TME and TTE [15]. However, in contrast to TTE, SCLN dissection is the most difficult part in TME because it is the deepest procedure from both the neck and abdomen, for which a detailed understanding of the surgical anatomy and a high level of skill specific to TME are essential. Regarding surgical anatomy, we previously performed a preoperative 3D-CT evaluation of the bronchial arteries for the mediastinal courses in the transcervical view to safely dissect the deep mediastinal lymph nodes, including SCLN, with the transcervical approach [11]. The present study evaluated the mediastinal shape and narrowness associated with the difficulty of transcervical SCLN dissection using preoperative 3D-CT images. In comparisons of easy and difficult cases, the transcervical inclination angle to the deep mediastinum (the cervical angle) and mediastinal narrowness (the aorta distance) were identified as significant anatomical predictors of difficulty, and the tumor location (middle thoracic tumor) was also identified as a significant predictor in the univariate analysis. Furthermore, the aorta distance and middle thoracic tumor were selected as independent predictors of difficulty in the multivariate analysis.

The mediastinum is a narrow space surrounded by the bilateral mediastinal pleura, sternum, thoracic spine, thoracic inlet, and diaphragm [16]. We empirically understand that the mediastinal shape or structures affect the difficulty of TME. In the transcervical procedure, the narrowness of the thoracic inlet is the first obstacle, with the projection of the clavicle head or an enlarged thyroid sometimes limiting device handling. The narrowness of the deep mediastinum is another obstacle, such that the aortic arch or left main bronchus needs to be appropriately avoided with the left-hand retractor. Avoidance of the aortic arch to the left expands surgical view for lymphadenectomy along the aortic arch, while lifting of the left main bronchus lengthens the distance between the tracheal carina and vertebra so as to expand surgical view for SCLN dissection. The right-hand device angle is also important for SCLN dissection. If the device is inserted square with the SCLN plane as much as possible, SCLN may be separated more easily from the tracheal carina. In the present study, middle thoracic tumor was also associated with the difficulty. Strong or extensive adhesion was often observed between advanced tumors of the middle thoracic esophagus and the surroundings, such as the left main bronchus or SCLN. In these cases, combined dissections with transcervical and transhiatal approaches were essential for safe and complete dissection. These clinical findings appear to be supported by the present results.

To the best of our knowledge, this is the first study to assess the technical difficulty of TME with a focus on transcervical SCLN dissection. Two previous studies have examined the factors influencing technical difficulty in esophagectomy [19, 20]. Fujiwara et al. identified the aorta-vertebra angle, sternum-vertebra distance, and clinical T stage as predictive factors for the technical difficulty of thoracoscopic esophagectomy in the left decubitus position [19]. Okamura et al. reported that the vertebral body projection at the middle thoracic part was a useful tool for predicting the difficulty of thoracoscopic esophagectomy in the prone position [20]. In these studies, the thoracic surgical time or blood loss was selected as the variable representing difficulty. In the present study, the difficulty was focused on SCLN dissection which represents the deepest procedure, but only a part among transmediastinal procedures, for which these surgical variables might be unsuitable. Then, the difficulty was judged by whether to add the transhiatal procedure for complete SCLN dissection, and was associated significantly with the variables derived from the mediastinal shape or narrowness, not with surgical variables.

There are some limitations. This was a retrospective study with small sample size in a single institute. although SCLN dissection was performed safely for all the study patients by a fixed team, judgements on whether to complete SCLN dissection with the transcervical approach may depend on the subjectivity or learning curve of the surgeon. In this regard, chronological distribution of difficult cases in relation to the aorta distance was investigated (Fig. 3). Consequently, the difficult cases were not concentrated in the early period, but rather increased in the later period, which suggests that transcervical SCLN dissection might be difficult in the narrow mediastinal cases even if the surgeon’s skill was improved, and the conversion to transhiatal approach might be judged appropriately according to the learning curve. In addition, the mediastinal shape or narrowness was only assessed for the width of the ventral–dorsal side. Narrowness in the lateral direction may also affect difficulty.

In conclusion, we defined new indicators using preoperative 3D-CT images to assess the difficulty of transcervical SCLN dissection, and the aorta distance and middle thoracic tumor were identified as independent predictive factors. SCLN dissection needs to be performed in combination with the transhiatal approach if difficulty is predicted, particularly for surgeons who introduce TME.

## Data Availability

The datasets used and/or analyzed during the present study are available from the corresponding author upon reasonable request.

## References

[1] Sung H, Ferlay J, Siegel RL (2021). Global cancer statistics 2020: GLOBOCAN estimates of incidence and mortality worldwide for 36 cancers in 185 countries. CA Cancer J Clin.

[2] Natsugoe S, Matsumoto M, Okumura H (2010). Clinical course and outcome after esophagectomy with three-field lymphadenectomy in esophageal cancer. Langenbeck’s Arch Surg.

[3] Fujiwara H, Shiozaki A, Konishi H (2019). Transmediastinal approach for esophageal cancer: a new trend toward radical surgery. Asian J Endosc Surg.

[4] Mori K, Yamagata Y, Aikou S (2016). Short-term outcomes of robotic radical esophagectomy for esophageal cancer by a nontransthoracic approach compared with conventional transthoracic surgery. Dis Esophagus.

[5] Fujiwara H, Shiozaki A, Konishi H (2017). Perioperative outcomes of single-port mediastinoscope-assisted transhiatal esophagectomy for thoracic esophageal cancer. Dis Esophagus.

[6] Mori K, Aikou S, Yagi K (2017). Technical details of video-assisted transcervical mediastinal dissection for esophageal cancer and its perioperative outcome. Ann Gastroenterol Surg.

[7] Fujiwara H, Shiozaki A, Konishi H (2016). Mediastinoscope and laparoscope-assisted esophagectomy. J Vis Surg.

[8] Tachimori Y, Ozawa S, Numasaki H (2016). Efficacy of lymph node dissection by node zones according to tumor location for esophageal squamous cell carcinoma. Esophagus.

[9] Udagawa H, Ueno M, Shinohara H (2012). The importance of grouping of lymph node stations and rationale of three-field lymphadenectomy for thoracic esophageal cancer. J Surg Oncol.

[10] Fujiwara H, Shiozaki A, Konishi H (2021). Transmediastinal approach for esophageal cancer: upper and middle mediastinal dissection with single-port technique. Atlas of minimally invasive techniques in upper gastrointestinal surgery.

[11] Maeda T, Fujiwara H, Konishi H (2022). Preoperative 3D-CT evaluation of the bronchial arteries in transmediastinal esophagectomy for esophageal cancer. Esophagus.

[12] Brierley JD, Gospodarowicz MK, Wittekind C (2017). TNM classification of malignant tumours.

[13] Japan Esophageal Society (2017). Japanese classification of esophageal cancer, 11th edition. Esophagus.

[14] Kitagawa Y, Uno T, Oyama T (2019). Esophageal cancer practice guidelines 2017 edited by the Japan Esophageal Society. Esophagus.

[15] Shibamoto J, Fujiwara H, Konishi H (2021). Evaluation of subcarinal lymph node dissection and metastasis in transmediastinal esophagectomy. Esophagus.

[16] Ugalde PA, Pereira ST, Araujo C (2011). Correlative anatomy for the mediastinum. Thorac Surg Clin.

[17] Hu W, Liang Y, Zhang S (2013). Impact of subcarinal dissection on short-term outcome and survival following esophagectomy. Am J Surg.

[18] Tang H, Tan L, Wang H (2019). Is routine subcarinal lymph node dissection necessary in superficial esophageal squamous cell carcinoma? A propensity score matching analysis. J Cancer.

[19] Fujiwara Y, Lee S, Gyobu K (2019). Predictive factors of difficulty of thoracoscopic esophagectomy in the left decubitus position. Esophagus.

[20] Okamura A, Watanabe M, Mine S (2016). Factors influencing difficulty of the thoracic procedure in minimally invasive esophagectomy. Surg Endosc.

